# Can the sports apps using play an intervening role in the process of college students’ exercise motivation affecting mental health by exercise adherence?

**DOI:** 10.1097/MD.0000000000040062

**Published:** 2024-10-11

**Authors:** Jun Li, Lingjie Wang, Feng Xia, Yuekun Shao, Xinyi Ma

**Affiliations:** a School of Design, Hainan Vocational University of Science and Technology, Haikou, China; b Hengshui University, Hengshui, China; c School of Marxism, Hainan University, Haikou, China; d Sports and Military Education Department, Hainan Vocational University of Science and Technology, Haikou, China; e Maritime College, Hainan Vocational University of Science and Technology, Haikou, China.

**Keywords:** exercise adherence, exercise motivation, mental health, sports apps using

## Abstract

The continuous increase in college students’ use of the Internet and their mental health problems caused by a sedentary lifestyle, lack of exercise, and obesity have gradually attracted researchers’ attention. There is still more work to be done to understand “how” and “under what circumstances” exercise motivation effects mental health, despite the fact that many of these studies have demonstrated that it has a significant impact on it. Four hundred forty-three college students in China were selected to participate in this study in order to explore the impact of exercise motivation on mental health. The mediating role of exercise adherence and the moderating role of sports apps using in this relationship. The results show that exercise motivation has a significant positive impact on college students’ mental health; exercise adherence partially mediates the relationship between exercise motivation and mental health; and the relationship between exercise motivation and exercise adherence is moderated by sports apps using. Specifically, the relationship between exercise motivation and exercise adherence is stronger for college students with high sports apps using. This study is based on the Internet using to promote physical exercise to college students, thereby alleviating their psychological problems caused by a sedentary lifestyle, lack of exercise, and obesity in the Internet era. New ideas are also provided for intervention in college students’ mental health.

## 1. Introduction

Today’s society is being negatively impacted by the serious public health and social issue of mental health (MH). As college students’ psychological and thought processes are still immature, They are more susceptible to MH issues.^[1]^ Researchers have found that the incidence of MH problems among college students (CS), the severity of symptoms, their use of college counseling services, and treatment time are all on the rise.^[2]^ Poor MH will not only make it hard for CS to focus on their studies, but it will also have a negative impact on their academic investment and performance, which is likely to lead to repeating grades and/or dropping out of school. More seriously, it will also affect their everyday life and health due to insomnia, depression, anxiety, suicide, etc.^[3]^ Therefore, scholars of pedagogy, psychology, and medicine have always focused on exploring the factors that influence MH.

Based on the previous academic achievement, the discussion of the factors that affect the MH of CS was focused on the external environment, such as family, school, and society.^[4,5]^ However, since the development of information technology and the change of modern lifestyle in recent years, CS have tended to spend more time using the Internet, and individuals’ MH problems caused by factors such as a sedentary lifestyle, lack of exercise, and obesity have gradually attracted scholars’ attention.^[6–8]^ Exercise psychologists believe that physical exercise is important for developing and effectively improving both physical and MH.^[9]^ Kilpatrick underscores that the most effective indicator for predicting physical exercise is exercise motivation,^[10]^ which is defined as the cause or perceived stimulation of individuals initiating or maintaining physical exercise, and is an important factor in relieving psychological stress. According to the self-determination theory, exercise motivation is an intrinsic form of motivation, which is generated by people’s desire to obtain happiness and satisfaction from physical exercise, and is closely related to their MH.^[11,12]^ Previous researchers have also shown that exercise motivation can positively predict MH. For example, Maltby and Day used a sample of 227 university students and found that exercise motivation had a significant and positive impact on their MH^[13]^; similarly, Rouse, Ntoumanis, Duda, Jolly, and Williams used a sample of 347 British adults and found that those had significantly better MH than individuals who were less driven to exercise.^[14]^

However, although previous theorists and researchers have shown that exercise motivation can predict MH, the process and conditions of its influence are still unclear. Furthermore, it has been shown in recent studies that one of the most important reasons for college students’ lack of exercise is their addiction to the Internet, but most of these studies have been focused on helping these students to rid themselves of their Internet addiction.^[15,16]^ However, in the digital age, expecting CS to avoid using the Internet is unreasonable. It is much more critical to skillfully use an Internet intervention to enhance their adherence to exercise to improve their MH, and sports apps have been shown to be a kind of software that positively promotes persistent physical exercise based on Internet technology.^[17]^ In addition, many researchers have confirmed that exercise motivation can effectively promote adolescents’ exercise adherence,^[18]^ which is a factor that has an extremely positive effect on their MH.^[19]^ In summary, exercise adherence and sports apps using may be a process and condition of the impact of exercise motivation on MH.

Hence, the first objective of this study is to investigate the mediating role of exercise adherence on the relationship between exercise motivation and MH by analyzing the elements that encourage CS to exercise. The additional objective of this study is to investigate the relationship between exercise motivation and college students’ MH, with Internet usage (using sports apps) serving as the moderating factor. The value of this study lies in its ability to integrate and expand previous theories and research and produce new ideas for college students’ MH intervention.

## 2. Literature review

### 2.1. Exercise adherence as a mediator

Exercise adherence is the behavioral tendency of individuals to persist in making an effort during physical exercise.^[20]^ According to the self-determination theory, individuals have strong physical exercise motivation because their exercise intention contains strong emotional components of self-investment and active participation. Hence, the intention to exercise for a longer time is higher, and the time to participate in exercise is longer.^[21]^ In their research, Rodrigues, Teixeira, Neiva, Cid, and Monteiro also pointed out that motivation for physical exercise can promote persistent exercise behavior and is an effective indicator for predicting adherence to exercise.^[22]^ Researchers have discovered that adolescents who pursue independence tend to have more persistent exercise behavior driven by spontaneous exercise motivation (under non-compulsory conditions).^[23]^ It has also been shown in empirical studies that exercise motivation can positively predict exercise adherence. For example, Kang et al found that exercise motivation had a significant and positive impact on exercise adherence,^[18]^ and the exercise frequency and duration of a sample of 1079 exercisers could be positively predicted by exercise motivation.^[24]^

Previous researchers have also demonstrated that exercise adherence affects the MH of CS.^[25]^ Firstly, from a neurobiology perspective, continuous physical exercise increases the secretion of dopamine and endorphins in the body by stimulating the sympathetic nerve and hypothalamus, thereby relieving tension and anxiety and promoting good MH.^[26]^ Secondly, based on an analysis of psychology and sociology, CS with good exercise adherence can establish close friendships with their peers during continuous physical exercise. They can also reduce their loneliness and improve their social ability based on mutual recognition. Sharing happiness can improve self-confidence, which, in turn, improves MH.^[27,28]^ When de Jonge-Heesen et al conducted semi-structured interviews with 15 CS who suffered from depression,^[29]^ they found that continuous physical exercise could effectively alleviate their mental illness. Likewise, numerous empirical research have demonstrated the major benefits of exercise on MH.^[7,19]^ Therefore, the first hypothesis as follows:

H1: Exercise adherence has a mediating effect between exercise motivation and MH.

### 2.2. Sports apps using as a moderator

According to the existing evidence, exercise motivation enables individuals to initiate and persist with exercise, but does not constitute a sufficient condition for forming exercise adherence behavior.^[30]^ Hence, there may be some moderating factors between exercise motivation and adherence. Although the persistence of college students’ physical exercise is still not optimistic under traditional intervention methods, new methods are urgently needed. Sports apps are gradually becoming widely used in health intervention due to their advantages of simple implementation, perfect functions, and easy portability.^[31]^ Hence, this study adopts sport apps as a moderating variable to examine the relationship between exercise motivation and exercise adherence.

The transtheoretical model of behavioral change has been validated for intervention in initiating and maintaining exercise behavior.^[32]^ The design strategy of most sports apps is based on this theoretical model. The initiation and maintenance of individuals’ sports behavior are improved by specific strategies, such as self-monitoring, goal setting and performance feedback in a concrete realization of the behavioral change transtheoretical model.^[33,34]^ Sports apps are third-party applications with functions such as recording and sharing sports data, and guiding sports learning.^[34]^ According to the expectancy-value model, sports apps can improve college students’ awareness of the effects and health risks of physical exercise with information about calorie consumption, data comparison before and after exercise, and various physical health indices, thereby increasing the likelihood that they will start and continue to maintain exercise behavior.^[35,36]^ From the perspective of hedonic motivation, although individuals will be driven by exercise motivation to adopt exercise behavior, whether that exercise behavior will persist for a long time depends on their experience of the exercise process. Those individuals who have a pleasant experience of the exercise process will be more inclined to persist in exercising for a long time, and those who have a unpleasant experience may stop exercising altogether.^[37,38]^ The functions of sports apps, such as sports sharing, sports social networking, and medal rewards, enable CS to have a positive experience of exercise, which may be transformed into a higher degree of exercise motivation, and then promote a higher level of exercise adherence.^[39,40]^

As empirical researchers also illustrate that sports apps can significantly promote individuals’ exercise adherence,^[41]^ it is speculated in this study that the sports apps using could favorable to enhance college students’ exercise motivation and exercise adherence. In other words, when CS are motivated to exercise, sports apps usings as an active intervention is likely to enhance the impact of exercise motivation on exercise adherence. Therefore, the second hypothesis is proposed as follows:

H2: Sports apps using has a moderating effect on exercise motivation and exercise adherence.

**Figure 1.**
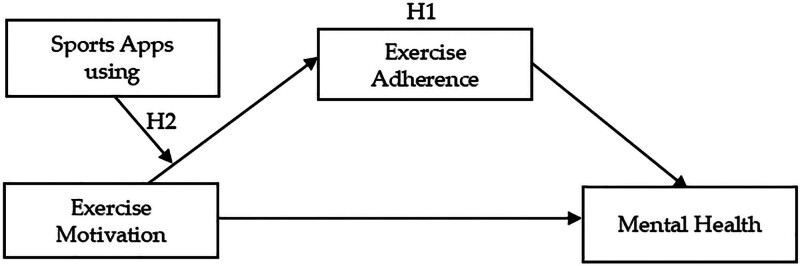
Hypothetical model.

In summary, as shown in Figure 1, a regulatory mediation model was constructed in this study to explore the mediating role of exercise adherence between exercise motivation and MH, and the moderating role of sports apps using between exercise motivation and exercise adherence. A moderating mediation model can provide a deeper understanding of the process and conditions of the impact of exercise motivation on MH than a single mediation model or a moderation model.^[42]^

## 3. Method

### 3.1. Participants and procedure

This study surveyed Chinese CS using a questionnaire using the convenience sampling technique. Participants who met the recruitment criteria were CS interested in the study’s topic of physical exercise and mental health and willing to participate. In order to mitigate potential information bias, the class head teachers responsible for distributing the questionnaires underwent professional training about the recruitment criterion and questionnaire entries. They were then tasked with recruiting CS and explaining the questionnaire entries to the participants. Furthermore, to guarantee participants’ comprehension of the questionnaire and accurate responses, the class head teachers delivered a concise explanation and instruction of the items before participants filled out the questionnaire. Additionally, participants were informed about the study’s objectives and the confidentiality agreement. They were assured that their questionnaires would be submitted and processed anonymously and that they had the option to decline participation or withdraw from the study at any point if they had any concerns. The class head teacher supervised the completion of the questionnaires. Participants could complete the online survey by scanning the QR code after giving their informed consent and signing the consent form, and the completed questionnaires were collected through the Questionnaire Start platform. Based on the above procedure, 553 questionnaires were distributed and collected in this study. After deleting invalid questionnaires, 443 valid questionnaires remained, with an effective recovery rate of 80.11%.

### 3.2. Research instruments

#### 3.2.1. Exercise Motivation Scale

The Exercise Motivation Scale compiled by Chen, Wang, Rong, Pan, and Bao,^[43]^ which has been found to possess high reliability and validity, was used in this study, with a sample of Chinese CS. The scale has 5 components: social motivation, ability motivation, fun motivation, appearance motivation, and health motivation. with 15 items, and a 5-point Likert scoring system (1 = not at all, 5 = very strong), with higher scores indicating stronger motivation for physical activity.

#### 3.2.2. Exercise adherence scale

The exercise adherence subscale of the physical exercise scale developed by Wu et al.^[38]^ It was also utilized in this investigation and proved to have great reliability and validity. The scale has 4 items, including a reversal question. A great deal of individuals have concerns about how reverse questions would affect the scale’s dependability. Researchers contend that by conducting some empirical and logical examination, the reverse questions can be eliminated.^[44]^ Therefore, this study deleted the reverse questions, and finally got the exercise adherence scale involve 3 items. The scale adopts a Likert 5-point scoring system (1 = disagree completely, 5 = agree completely); the higher the score, the better the exercise adherence.

#### 3.2.3. Sports apps using scale

The sports apps using scale compiled by S. Wang et al,^[41]^ which has also been proved to have high reliability and validity, was developed in this study to suit the Chinese cultural environment. The scale includes 4 dimensions of display interaction, follow-up improvement, intuitive feedback and goal management. There was a total of 12 items and the scale adopted a 5-point Likert scoring system (1 = completely disagree, 5 = completely agree); the higher the score, the higher sports apps using.

#### 3.2.4. Mental Health Scale

The Mental Health Scale compiled by the Whoqol Group is adopted in this study.^[45]^ The Chinese version of this scale has shown good reliability and validity among Chinese CS.^[46]^ The scale contains 6 items, including 1 reverse question. Similarly, according to the treatment of reverse questions in previous studies, this study deleted the reverse questions,^[44]^ and finally retained 5 items, and adopts a 5-point Likert scoring system (1 = disagree completely, 5 = agree completely); the higher the score, the higher the MH level.

### 3.3. Statistical analyses

Data collected for this study were analyzed using SPSS 21.0 and AMOS 25.0. First, the scale’s validity and reliability were examined using a reliability analysis and a confirmatory factor analysis. Second, the level of correlation between the variables and the total performance of the participants in each variable were analyzed using a descriptive analysis and Pearson correlation analysis. Thirdly, the SPSS PROCESS programme was used to test the moderating mediation model, and analyze the mediating effect of exercise adherence and the moderating effect of sports apps using. Finally, the Bootstrap method was used to further test the mediating effect of exercise adherence to correct the percentage of nonparametric bias, and a simple slope analysis was used to test the moderating effect of the further sports apps using.

## 4. Results

### 4.1. Reliability and validity

Results of the reliability analysis revealed that that the Cronbach *α* values of the 4 scales of exercise motivation, exercise adherence, sports apps using, and MH were 0.947, 0.908, 0.960, and 0.907, respectively. As they all reached a standard >0.700, the 4 scales all showed good reliability.^[47]^ In order to assess the reliability of the 4 scales: exercise motivation, exercise adherence, sports apps using, and MH, a multi-factor oblique model was developed. The model’s fitness was first evaluated using a confirmatory factor analysis, and the results showed that = 3.498 (<5); SRMR = 0.054, RMR = 0.061, and RMSEA = 0.075. These 3 indicators all reached a standard of <0.080. TLI = 0.908 and CFI = 0.922, which both reached a standard >0.900. GFI = 0.808, hence reaching a standard >0.800, and PGFI = 0.648, reaching a standard >0.5. All of this indicates a good model fit.^[48]^ Second, in Table 1, each scale item’s standardized factor loadings ranged from 0.774 to 0.966, all >0.500; each factor’s combined reliability values ranged from 0.881 to 0.956, all >0.600; and each factor’s average variance extraction value, between 0.661 and 0.879, all >0.400, indicating that the model had good convergent validity.^[49]^

**Table 1:** CFA of the exercise motivation.

Dimension	No.	SFL	CR	AVE
Health motivation	1	.793	.915	.782
	2	.948		
	3	.905		
Appearance motivation	1	.906	.942	.843
	2	.960		
	3	.887		
Fun motivation	1	.674	.900	.754
	2	.966		
	3	.935		
Ability motivation	1	.941	.922	.798
	2	.945		
	3	.785		
Social motivation	1	.875	.943	.847
	2	.962		
	3	.922		
Exercise adherence	1	.865	.911	.773
	2	.911		
	3	.860		
Show interaction	1	.828	.902	.754
	2	.854		
	3	.920		
Follow and improve	1	.808	.881	.713
	2	.849		
	3	.874		
Intuitive feedback	1	.894	.913	.779
	2	.922		
	3	.829		
Target management	1	.914	.956	.879
	2	.958		
	3	.940		
Mental health	1	.774	.907	.661
	2	.790		
	3	.836		
	4	.851		
	5	.813		

Additionally, a common method deviation test was carried out in this study using Harman single-factor test. All the variables were subjected to an unrotated principal component factor analysis, and 6 factors were identified with characteristic roots greater than one. The first factor could only explain 42.763% of the variation, which is less than the critical standard value of 50%, indicating that the common method bias problem in this study was not serious.^[50]^

### 4.2. Descriptive statistics and correlations

The 4 variables of exercise motivation, exercise adherence, sports apps using, and MH were subjected to a descriptive statistical analysis and correlation analysis, the results of which are shown in Table 2. Exercise motivation and exercise adherence were significantly positively correlated (*R* = 0.489, *P* < .001), exercise motivation and sports apps using were significantly positively correlated (*R* = 0.490, *P *< .001), exercise motivation and MH were significantly positively correlated (*R* = 0.377, *P* < .001), exercise adherence and sports apps using were significantly positively correlated (*R* = 0.655, *P* < .001), exercise adherence and MH were significantly positively correlated (*R *= 0.374, *P *< .001), and sports apps using was significantly positively correlated with MH (*R* = 0.382, *P* < .001). The correlation coefficients between 2 variables ranged from 0.374 to 0.655, indicating that there was low-to-medium correlation among the variables, and there was no serious collinearity problem.^[51]^

**Table 2:** Descriptive statistics and correlations.

Variable	M	SD	Exercise motivation	Exercise adherence	Sports apps using	Mental health
Exercise motivation	3.584	0.854	1			
Exercise adherence	3.157	0.915	0.489***	1		
Sports apps using	3.348	0.786	0.490***	0.655***	1	
Mental health	3.786	0.874	0.377***	0.374***	0.382***	1

*** *P* ＜ .001.

### 4.3. Mediating effect of exercise adherence

The SPSS PROCESS Model 4 was used in this study to test the mediating effect of exercise adherence, and the results are shown in Table 3. According to Model 1, exercise motivation has a significant positive effect on MH (B = 0.386, *P* < .001), while based on Model 2, exercise motivation has a significant positive effect on exercise adherence (B = 0.524, *P* < .001). After adding exercise adherence as an intermediary variable to Model 3, exercise motivation was still found to have a significant positive effect on MH (B = 0.261, *P* < .001). However, the influence was reduced compared with Model 1, and exercise adherence was also shown to have a significant positive effect on MH (B = 0.239, *P* < .001). The results indicated that exercise adherence is a partial mediator between exercise motivation and MH. The results of the further use of the bias-corrected nonparametric percentile Bootstrap method and random repeated sampling 5000 times to test the mediation effect, show that the direct effect value = 0.261, the 95% confidence interval (CI) does not contain 0 (LLCI = 0.162, ULCI = 0.360), the indirect effect value = 0.125, the 95% confidence interval does not contain 0 (LLCI = 0.072, ULCI = 0.185), the total effect value = 0.386, and the 95% confidence interval does not contain 0 (LLCI = 0.297, ULCI = 0.475). Therefore, mediation accounted for 32.383% of the total effect.

**Table 3:** Testing the mediation model of exercise adherence.

Variable	Model 1 Mental health Β (t)	Model 2 Exercise adherence Β (t)	Model 3 Mental health Β (t)
Exercise Motivation	0.386 (8.551***)	0.524 (11.773***)	0.261 (5.185***)
Exercise Adherence			0.239 (5.071***)
*R*²	0.142	0.240	0.190
F	73.113***	138.601***	51.464***

*Note*: B = unstandardized coefficients.

*** *P* ＜ .001.

### 4.4. Moderated mediation model

The SPSS PROCESS Model 7 was used to test the regulatory mediation model constructed in this study, and the results are shown in Table 4. The interaction term between exercise motivation and sports apps using had a significant positive effect on exercise adherence (Β = 0.116, *P* < .01), which indicates that sports apps using moderates the impact of exercise motivation on exercise adherence.

**Table 4:** Testing the moderated mediation model.

Variable	Model 1 Exercise adherence Β (t)	Model 2 Mental health Β (t)
Exercise motivation	0.235 (5.544***)	0.261 (5.185***)
Sports apps using	0.635 (13.752***)	
Exercise motivation × sports apps using	0.116 (2.908**)	
*R*²	0.476	0.190
F	133.165***	51.464***

*Note*: B = unstandardized coefficients.

** *P* ＜ .01.

*** *P* ＜ .001.

**Figure 2.**
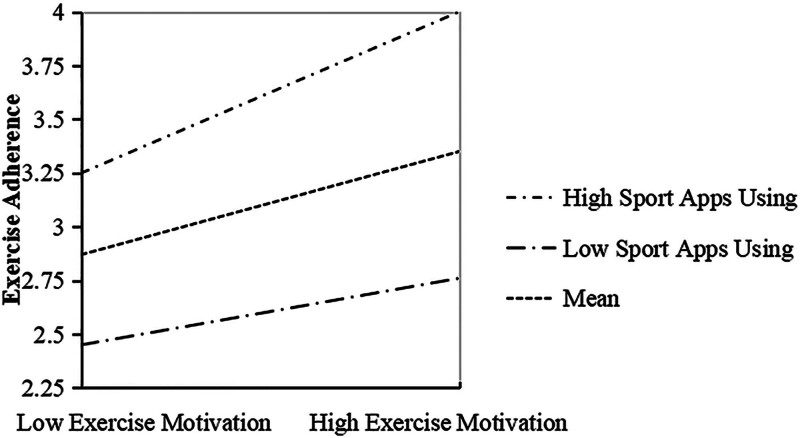
Moderating effect of sports apps using.

A simple slope analysis was used in this study to explain the moderating effect of using sports apps. As shown in Figure 2, the correlation between exercise motivation and exercise adherence is stronger for CS with higher sport apps using (simple slope = 0.326, *t* = 6.219, *P *< .001) than those who use fewer sports apps (simple slope = 0.144, *t* = 2.723, *P* < .01). This shows that the exercise motivation of CS who use sports apps is more likely to be transformed into exercise adherence than the exercise motivation of those who do not use sports apps. A bias-corrected nonparametric percentile Bootstrap method was further used to verify the moderation effect by random repeated sampling 5000 times. The results showed that the index of moderated mediation was 0.028 (LLCI = 0.009, ULCI = 0.055), and the confidence interval did not include 0, indicating a significant moderation effect.

## 5. Discussion

Inspired by previous theorists and researchers, this study was based on examining whether exercise adherence mediates the relationship between exercise motivation and the MH of CS and whether sports apps using also moderates this relationship.

Firstly, exercise adherence is a partial mediator between the exercise motivation and MH of CS. Although previous researchers showed that exercise motivation affects MH,^[13,14]^ the mediating mechanisms behind this association were unclear. However, the mechanism that connects exercise motivation and MH was identified in this study as a crucial mediation pathway that helps to clarify the connection between these 2 entities. Firstly, exercise motivation has a significant effect on exercise adherence, consistent with past studies’ findings.^[18,22]^ Based on self-determination theory, driving by exercise motivation (intrinsic motivation), CS desire to obtain psychological and physical pleasure from exercise, as well as improve their social skills.^[52,53]^ This intrinsic motivation will drive CS to actively participate in exercise and persist in doing so for a long time.^[21]^ Meanwhile, frequent exercise has been shown to have a large and beneficial effect on MH, which is consistent with previous studies.^[7,19]^ From a neurobiological perspective, long-term exercise promotes the body to secrete more dopamine and endorphins, alleviating psychological problems, such as tension and anxiety.^[26]^ In addition, during long-term physical exercise, CS and their peers enhance their social skills and self-confidence, and improved physical fitness improves MH.^[27,28]^ Therefore, when intervening in the MH of CS, it is not only necessary to stimulate the exercise motivation of CS but also to pay attention to their exercise persistence and train CS to develop the habit of exercise adherence to maintain stable mental health status.

Secondly, this study shows that sports apps using moderated the first half of the mediation relationship. According to the facilitation hypothesis in the “Protector-Protector Model,” 1 protector promotes the predictive effect of another protector on an outcome variable Positive emotions,^[54]^ especially the enjoyment conferred on by multimedia tools, significantly contribute to the promotion of a relationship between exercise motivation and exercise adherence,^[38,55]^ while the display interaction of sports apps, multimedia functions such as follow-up improvement, intuitive feedback and goal management, enable CS to enjoy a positive exercise experience.^[37,56]^ It may be that sports apps using will enhance college students’ exercise motivation and be the reason for its positive impact. This relationship can also be explained by the expectation-value model. Sports apps can intuitively show the changes in physical health before and after exercise, and can set different exercise goals for different people. In this way, CS can perceive the value of changes engendered by exercise, improve their confidence in completing exercise goals, and ultimately enhance the transformation of exercise motivation into exercise adherence.^[35,36]^ It is conceivable to witness a simultaneous effect of sports app use, exercise motivation, and exercise adherence on college students’ MH, hence, they must be considered at the same time, rather than in isolation, in order to successfully intervene in college students’ MH.

Additionally, it is interesting that in the age of the Internet, emphasis has gradually been paid to the psychological issues that CS have due to a sedentary lifestyle and a lack of exercise. with the rapid development of online games and short video social software.^[16,57]^ However, most previous academic achievements were solely focused on how to prevent CS from overusing the Internet to intervene in their psychological problems.^[15,58]^ However, in the digital age, expecting CS to avoid using the Internet is unreasonable. However, it is unrealistic to expect CS not to use the Internet in the Internet age. It is more important to consider how to improve their motivation to exercise, and even use the Internet skillfully to promote their persistence in exercising and then intervene in their MH. Based on the transtheoretical model of behavioral change, sports apps were creatively utilized in this study as a moderating variable, and to explore the mediating role of exercise adherence between exercise motivation and MH, and it was found that sports apps using enhances the effect of exercise motivation on MH through exercise adherence. In conclusion, this research contributes to our understanding of how physical activity affects MH, but it also helps to integrate and expand previous theories and research. It also provides new ideas for intervention in college students’ MH and lays a foundation for future research in this field.

Finally, it is essential to highlight that the theoretical model developed in this study can potentially serve as a reference for psychological intervention for overweight college students. Adolescent overweight and obesity rates have increased in recent years. Obesity not only impacts physical health but also leads to psychological issues. Adolescents with obesity often experience low self-esteem, depression, anxiety, interpersonal sensitivity, and other negative emotions due to body image concerns.^[59]^ The sedentary lifestyle and lack of exercise are significant contributors to obesity among CS.^[8]^ Therefore, addressing college students’ exercise adherence and reducing their body weight is crucial in tackling this issue. This study validated the promoting effect of internal motivation, specifically exercise motivation, on exercise adherence by self-determination theory. The findings of this study offer a theoretical reference for harnessing motivation to enhance college students’ commitment to physical exercise. Furthermore, in the age of mobile Internet, the information regarding physical exercise and health found on online platforms has an impact on the health cognition and behavior of obese individuals.^[60]^ For instance, an investigation into the impact of podcasts on users’ physical health found that podcasts focusing on weight loss induce physiological arousal and improve users’ understanding of physical exercise and health, thus effectively motivating users to engage in physical exercise and enhancing their overall physical and mental well-being.^[61]^ The sports apps examined in this study not only enhance users’ understanding of health by providing information on physical exercise and health but also encourage users to accomplish exercise goals by incorporating enjoyable aspects such as prizes for achieving objectives.^[35]^ Hence, the theoretical model developed in this study has the potential to enhance physical exercise and mental well-being among CS by addressing exercise motivation and health cognition and offers a more comprehensive basis for psychological interventions targeting obese CS.

## 6. Limitations

All studies have limitations and this 1 is no exception. Firstly, all the data was derived from college students’ self-reports, which may introduce social desirability or recall bias. Thus, to ensure that these biases did not severely influence the study’s findings, 2 strategies were implemented: precluding it by careful study design and evaluating it using statistical analysis, as advised by Podsakoff et al (2003).^[62]^ Even though Harman one-factor test revealed no serious problem of common method bias indicating the robustness of the results, future researchers are encouraged to use multiple research methods, such as interviews and observations, to reduce the inherent effects of any single method bias. Secondly, cross-sectional data was used for this study, which can only show the relationship between variables at specific points in time. Although some scholars believe that cross-sectional research can provide valuable information to reveal the relationship between variables,^[63–65]^ to confirm the findings of this study using longitudinal data, additional study is required. it was found in this study that exercise adherence partially mediates exercise motivation and MH, indicating that there may be other mediating factors in this relationship. And the quality of life is also an effective predictor of MH.^[66]^ Therefore, future researchers can explore this, or other possible mediating factors. In addition, since the means of Internet promotion based on sports apps using were only examined in this study, future researchers could explore or integrate multiple moderating factors in other fields.

## 7. Conclusion

In summary, exercise adherence was used as the mediating variable in this study and sports apps using as the moderating variable to explore the relationship between exercise motivation and the MH. The study found that the mediating effect of exercise adherence explains that exercise motivation can positively predict college students’ psychological well-being. sports apps using enhances the effect of exercise motivation on MH through exercise adherence. Due to the use of a network app platforms to encourage college students’ physical activity in an effort to address their psychological issues triggered on by a sedentary lifestyle and a lack of exercise in the Internet age, which is the one of the significances in this studies. The research results not only help to understand the underlying mechanism between exercise motivation and MH but also provide a new idea for a college students’ MH intervention.

## Acknowledgments

Thanks to all the participants in this study.

## Author contributions

**Conceptualization:** Jun Li, Lingjie Wang, Xinyi Ma.

**Data curation:** Xinyi Ma.

**Formal analysis:** Jun Li.

**Methodology:** Lingjie Wang, Yuekun Shao.

**Supervision:** Feng Xia.

**Software:** Xinyi Ma.

**Writing – original draft:** Jun Li, Lingjie Wang.

**Writing – review & editing:** Jun Li, Lingjie Wang, Feng Xia, Yuekun Shao, Xinyi Ma.
